# Interhemispheric neural characteristics of noxious mechano-nociceptive stimulation in the anterior cingulate cortex

**DOI:** 10.3389/fncir.2023.1144979

**Published:** 2023-05-05

**Authors:** Amir Aminitabar, Mahnoosh Mirmoosavi, Mohammad Taghi Ghodrati, Vahid Shalchyan

**Affiliations:** Neuroscience and Neuroengineering Research Laboratory, Department of Biomedical Engineering, School of Electrical Engineering, Iran University of Science and Technology, Tehran, Iran

**Keywords:** mechanical nociceptive pain, anterior cingulate cortex (ACC), local field potential (LFP), von Frey filament, evoked potential (EP), bilateral analysis

## Abstract

**Background:**

Pain is an unpleasant sensory and emotional experience. One of the most critical regions of the brain for pain processing is the anterior cingulate cortex (ACC). Several studies have examined the role of this region in thermal nociceptive pain. However, studies on mechanical nociceptive pain have been very limited to date. Although several studies have investigated pain, the interactions between the two hemispheres are still not clear. This study aimed to investigate nociceptive mechanical pain in the ACC bilaterally.

**Methods:**

Local field potential (LFP) signals were recorded from seven male Wistar rats’ ACC regions of both hemispheres. Mechanical stimulations with two intensities, high-intensity noxious (HN) and non-noxious (NN) were applied to the left hind paw. At the same time, the LFP signals were recorded bilaterally from awake and freely moving rats. The recorded signals were analyzed from different perspectives, including spectral analysis, intensity classification, evoked potential (EP) analysis, and synchrony and similarity of two hemispheres.

**Results:**

By using spectro-temporal features and support vector machine (SVM) classifier, HN vs. no-stimulation (NS), NN vs. NS, and HN vs. NN were classified with accuracies of 89.6, 71.1, and 84.7%, respectively. Analyses of the signals from the two hemispheres showed that the EPs in the two hemispheres were very similar and occurred simultaneously; however, the correlation and phase locking value (PLV) between the two hemispheres changed significantly after HN stimulation. These variations persisted for up to 4 s after the stimulation. In contrast, variations in the PLV and correlation for NN stimulation were not significant.

**Conclusions:**

This study showed that the ACC area was able to distinguish the intensity of mechanical stimulation based on the power activities of neural responses. In addition, our results suggest that the ACC region is activated bilaterally due to nociceptive mechanical pain. Additionally, stimulations above the pain threshold (HN) significantly affect the synchronicity and correlation between the two hemispheres compared to non-noxious stimuli.

## 1. Introduction

According to the definition provided by the International Association for the Study of Pain (IASP), “An unpleasant sensory and emotional experience associated with, or resembling that associated with actual or potential tissue damage.” Although pain is a sensory response that is activated to protect the individual from injury, in the long term, it can become a debilitating condition (Cox et al., 2006). One of the well-known pain categorization methods was introduced by Woolf (2010). According to this definition, pain is divided into three categories: nociceptive, inflammatory, and pathological. Nociceptive pain acts as a physiological protective mechanism with early warning, which is essential for detecting and minimizing contact with harmful pain stimuli. Inflammatory pain has an adaptive and protective role which helps to heal the injured limb by preventing contact and movement. The third category is pathological pain which has no protective function and is a kind of incompatibility with the abnormal functioning of the nervous system (Woolf, 2010). The ability to assess the intensity of painful stimuli is a major property of the nociceptive system. Nociception is a process in which thermal, mechanical, and chemical stimuli are detected by a group of peripheral nerves called nociceptors (Dubin and Patapoutian, 2010). Due to their biophysical and molecular properties, pain receptors are activated only when the intensity of the stimulus exceeds the pain threshold (Woolf, 2010). This type of pain resolves after tissue healing or the absence of harmful stimuli (Basbaum et al., 2009; Larson et al., 2019).

Pain includes sensory and affective aspects. The sensory aspect of pain includes duration, intensity, and type of pain. Affective pain is the emotional and unpleasant aspects of pain, including fear, worry, and anxiety. Two different pathways process the sensory and affective aspects of pain (Johansen and Fields, 2004; Basbaum et al., 2009; Navratilova et al., 2012; Zhang et al., 2017; Zhou et al., 2018; Larson et al., 2019). In the sensory pathway, the thalamus, primary somatosensory cortex (S1), secondary somatosensory cortex (S2), and anterior cingulate cortex (ACC) process the signals received from the spinal cord. Whereas in the affective pathway, the thalamus, insula, amygdala, prefrontal cortex, and ACC are involved in processing the emotional aspect of pain (Xiao and Zhang, 2018). Ascending nociceptive pain pathways for processing the sensory and affective pain terminate at S1 and ACC, respectively (Lubar, 1964; Melzack and Wall, 1965; Foltz and White, 1968; Melzack and Casey, 1968; Talbot et al., 1995; Koyama et al., 2000; Johansen et al., 2001; LaGraize et al., 2006). ACC areas are getting more attention in the pain assessment field since it encodes both aspects of pain. Studies on humans have shown that the ACC also measures nociceptive pain intensity (Coghill et al., 1999; Büchel et al., 2002). In addition, several studies using *in vivo* recordings in rats have shown that specific neurons in the ACC convey information about the intensity and onset of pain (Chen et al., 2017). Electrophysiological recordings in rabbits and rats showed that nociceptive neurons in the ACC had large, bilateral receptive fields (Sikes and Vogt, 1992; Yamamura et al., 1996; Vogt and Sikes, 2000). In this study, ACC was chosen to investigate the sensory aspect of pain and specifically to discriminate pain intensities. The LFP signals recorded from the ACC region were used to assess pain, as intracortical signals, at the mesoscopic and macroscopic levels supply valuable physiological information for depicting pain at an acceptable timescale comparable with single neural activity (Peng et al., 2018; Ploner and May, 2018).

Most studies that examined the neuronal response of nociceptive pain in the ACC region used thermal stimuli and investigated neuronal responses at the spike and LFP levels. In a study by Li et al. (2017). recording electrodes were implanted in the ACC, orbitofrontal cortex, S1, and periaqueductal gray. Local field potential (LFP) patterns in these regions were investigated by applying noxious and non-noxious thermal stimulation. They concluded that alpha and beta power decreased after noxious stimulation and gamma power increased. Also, the two stimuli were separated with 86% accuracy (Li et al., 2017). In a similar study, recording electrodes were implanted in the S1 and ACC. The results showed that EPs are synchronous in two regions; however, their amplitude is different. It was also reported that the amplitude of EPs was directly related to the stimulation intensity and the phase-amplitude coupling (theta phase vs. gamma amplitude) was stronger in the S1 than in the ACC (Xiao et al., 2019). In another study, brain signals were recorded from S1, ventral posterior lateral thalamic nuclei, medial dorsal thalamic nuclei, and ACC. Laser stimulation was used to cause pain. They observed that noxious stimuli have double-peak evoked potentials (EPs) in all regions, and there is a significant correlation between stimulation intensity and the number of responsive neurons and firing rates (Zhang et al., 2011). In another study by Zhang et al. (2018) electrodes were implanted in the ACC regions, and thermal stimulation (laser) was used to cause pain. Stimuli were applied with three intensities: high-intensity noxious, low-intensity noxious, and non-noxious. The classification results were as follows: high-intensity noxious vs. non-noxious with 80% accuracy, non-noxious vs. low-intensity noxious with 65% accuracy, and high-intensity noxious vs. low-intensity noxious with 65% accuracy were separated. Theta and high gamma bands were also shown to be the best bands for pain intensity classification. In addition, LFP features were better than spike features, but combining these two features improved the classification results (Zhang et al., 2018).

**FIGURE 1:**
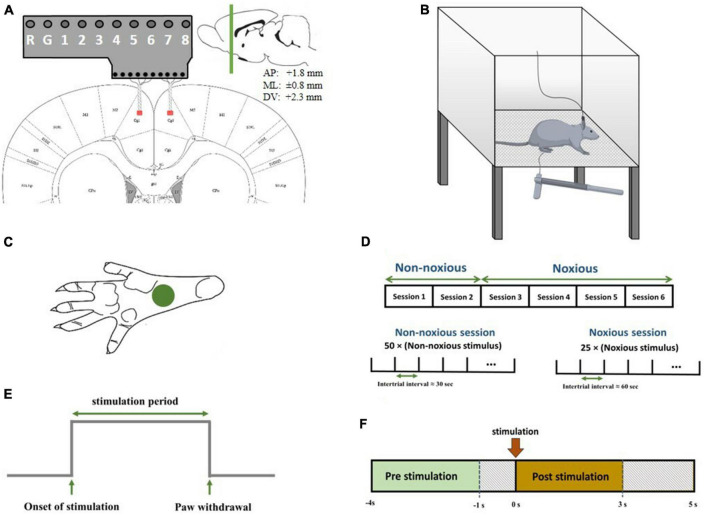
**(A)** Schematic diagram representing electrode implantation. The signals were recorded from areas denoted by red patches. Electrodes were implanted symmetrically in both hemispheres. The coordinates of the recording regions are +1.8 mm anterior/posterior, ±0.8 mm medial/lateral, and 2.3 mm dorsal/ventral. **(B)** Schematic diagram of electrophysiological recordings in freely moving rats. Each trial of peripheral stimulation lasted either 5s in the case of no withdrawal (non-noxious stimulus or NS). **(C)** The correct area for mechanical stimulation. **(D)** The structure of recording sessions. Recording sessions were held on six consecutive days, with one recording session each day. NN stimulation was applied in the first two sessions, and HN stimulation was applied in the subsequent four sessions. The structure of NN and HN sessions is shown at the bottom. **(E)** A sample output signal of the von Frey device, in which the stimulation-related parameters are continuously sent to the recording device. **(F)** The interval of pre-stimulation and post-stimulation to extract features.

Nociceptive mechanical pain is more common in daily life compared to other sorts of pain, so investigating this type of pain is important. To the best of our knowledge, the LFP response to painful mechanical stimulation has not been studied bilaterally in the ACC region. The first purpose of this study is to investigate the similarities and differences in the neuronal response to mechanical stimuli with high-intensity noxious (HN) and non-noxious (NN) intensities at the LFP level. The effect of frequency bands and time intervals on classification accuracy was also examined. The persistence of the pain effect is another issue that was examined. In this work, microelectrodes were implanted bilaterally in the ACC regions (Figure 1A). The second purpose of this study is to scrutinize the ACC in two hemispheres. To this end, evoked potential (EP), power, correlation, and PLV of LFP responses between two hemispheres were analyzed at five-second intervals after stimulus onset.

## 2. Materials and methods

### 2.1. Animals and surgery

Seven male adult Wistar rats weighing 320 ± 38 g were used in this study. Rats were kept under standard conditions of a 12-h dark-light cycle with free access to water and food. The animals were given an average of 10 days to adapt to the new environment before the experiment began.

A mixture of ketamine (100 mg/kg) and xylazine (10 mg/kg) was used to anesthetize the rats (Wang et al., 2008; Khorasani et al., 2016). The depth of anesthesia was checked during surgery using a pinch test. A total of 20 mg of ketamine was injected intraperitoneally if the depth of anesthesia decreased. After incising the skin over the surgical regions, identifying the lambda and bregma, and determining the location of the holes to reach the ACC, the implantation site was perforated, and the microelectrodes were gently inserted into the regions. Microelectrodes were implanted in the ACC of both hemispheres. The coordinates of the regions were determined according to the rat brain map (Paxinos and Franklin, 2019) (+1.8 mm anterior/posterior, ± 0.8 mm medial/lateral, and 2.3 mm dorsal/ventral) (Figure 1A).

Advent tungsten microelectrodes were used to record cortical signals, and their diameter without insulation cover was 25 micrometers. Due to their small diameter and high flexibility, these microelectrodes cannot penetrate the brain; therefore, four microelectrodes were twisted together to increase their strength for penetration into the cerebral cortex. The connection of microelectrodes was checked after insertion, and then the electrodes were covered by dental cement. After surgery, each rat was allowed to recover with free access to food and water for 1 week in a separate cage, and signal recordings were done after this recovery period. Analgesia after surgery was not used during the recovery period, because using analgesic drugs increases the risk of interfering with the results (Benrath et al., 2004).

The local ethics committee (The animal care and use committee of Neuroscience and Neuroengineering Research Laboratory, Iran University of Science and Technology) approved all the issues, including anesthetization, craniotomy surgery, and recovery procedures, and all the procedures were carried out in compliance with ARRIVE guidelines on the animal.^1^

### 2.2. Mechanical stimulation

In recording sessions, rats were placed in a Plexiglas chamber with one transparent and three opaque sides so that the experimenter could observe the behavior of the rat. The chamber floor had a lattice structure so the experimenter could easily pass the von Frey filament through the holes and mechanically stimulate the rat’s hind paw. The diameter of each hole was 2 mm, and the distance between the holes was 5 mm. The upper surface of the cage was open to allow the rat to move easily (Figure 1B).

There are several methods for measuring the sensitivity to mechanical stimulation. This study used the von Frey method, which is the gold standard method for mechanical stimulation. Hand-made von Frey filaments were used to apply mechanical stimulation to the rat’s hind paw (de Sousa et al., 2014). Stimuli were applied with high-intensity noxious (HN) and non-noxious (NN) intensities. The diameters of the hand-made von Frey filaments were equal, and, as the intensity of stimulation is inversely related to the length of the filament, filaments with different lengths were used to change the force applied. Stimulation with intensities of 8 and 30 g was used in this study.

An electrical device using a load cell, analog-to-digital converter, and microcontroller was developed to record the onset of stimulation and paw withdrawal accurately. The signal from the load cell was converted to a digital signal using an analog-to-digital converter and sent to the signal acquisition device by the microcontroller. This device was powered with a battery pack to avoid interference with brain recordings. The output of the device was a digital signal that contains information about the stimulation onset time, stimulation period, and the time of paw withdrawal (Figure 1E). Multi-channel systems (ME16-FAI-μPA-System) were used to record the data. Microelectrodes were attached to the eight-channel preamplifier, and LFP signals were recorded at a sampling rate of 1,000 Hz.

### 2.3. Experiment protocol

Before the surgery, the rats were placed in the chamber to evaluate their behavioral response to mechanical stimulation and make them accustomed to the recording environment. In main recording sessions, first, the rat was placed in the recording chamber, and the signals were recorded after the animal got acquainted with the recording environment. Six sessions of stimulation and simultaneous recording of brain signals were held for each rat. In the first two sessions, NN stimulations (8 g) were applied to the left hind paw, and HN (30 g) stimulations were administered in the subsequent four sessions. Our rationale for this arrangement in the recording sessions was the emotional aspect of pain in the ACC region. Fifty stimuli were applied to the left hind paw in each NN recording session, in which the interval between them was 30 s. A total of 10 to 15 stimuli were applied at the beginning of each session to eliminate fear, worry, and anxiety in NN sessions. In HN recording sessions, 25 stimuli were applied with approximately 60-s intervals between two consecutive stimuli (Figure 1D). All stimuli were applied to the left hind paw (Figure 1C).

### 2.4. Pre-processing

The signal was filtered by a 4th-order Butterworth band-pass filter with (3–350) Hz cutoff frequency to extract LFP information. Also, the mains artifact and its harmonics were removed with a notch filter. To eliminate motion artifacts, oscillations less than 3 Hz in the preprocessing step were removed. In addition, trials distorted by motion artifacts were detected and removed using the Grubbs algorithm (Statistics, 2013). Grubbs is an iterative algorithm that removes one outlier per iteration based on hypothesis testing. Trials in which more than 10% of samples were identified as outliers were excluded. Also, the trials in which the behavioral response of the rat was long-term were considered defective trials. Given the non-stationary nature of brain signals and the recording environment, the signal amplitude in each recording session could be different across sessions. Therefore, the signal of each channel in each session was normalized. The z-score method, which was used for normalization, can be formulated as follows (Cohen, 2019):


(1) $$z\,\left(t\right)=\frac{x\,\left(t\right)-\bar{\text{x}}}{\sigma }$$

In this equation, x(t) is the LFP signal of each recording channel, σ is the standard deviation, $\bar{\text{x}}$ is the signal mean in one session, and z (t) is the normalized LFP signal.

Channels that were noisy or had abnormal mean and standard deviation were identified and removed from further analysis. In total, one channel from rat 6 and one channel from rat 7 was removed. The LFP signals recorded from the brain demonstrate the local activities of neurons within the range of 200–400 μm from implanted microelectrodes (Katzner et al., 2009; Xing et al., 2009). Due to the fact that in our study, the microelectrodes were implanted close to each other (30–50 μm), the LFP signals recorded by close electrodes shared a significant amount of data. Thus, non-noisy recorded channels from each hemisphere were averaged and used for further analysis. All data processing was carried out with MATLAB 2020b (The MathWorks, Inc., USA).

### 2.5. Evoked potentials (EPs)

Evoked potentials (EPs) in LFPs show the activity of numerous neurons in response to a stimulus (Bradley and Keil, 2012). EPs are associated with the low-frequency activity of neurons and are often extracted by averaging the time-aligned signal over several trials (Garcia-Larrea et al., 2002; Xiao et al., 2019). In this study, EPs related to HN and NN stimulation were examined. To investigate the variations in the EPs pattern, the parameters of the time interval between the stimulation onset and first positive peak (P1), the time interval between the stimulation onset and first negative peak (N1), and the amplitude of P1 and N1 were extracted. Wilcoxon test was used to evaluate the significance between the extracted parameters.

### 2.6. Spectrum analysis

Given that brain signals are non-stationary, the spectral analysis of these signals is mainly done using time-frequency analysis. Continuous wavelet transform (CWT) is a widely-used time-frequency analysis method and can demonstrate the variations in the spectrum over time (Bu, 2007). Scalogram figures were used to examine power over frequency and time. Scalogram is a representation of a wavelet transform, having axes for time, scale, and coefficient values. According to the similarity of brain signals to the Morlet mother wavelet (Herrmann et al., 2014), the Morlet wavelet transform was used to display the scalogram. The power was normalized to the baseline using the z-score method to express the signal power spectrum variations more accurately (Cohen, 2019).

### 2.7. Classification

The classification of the presence and the intensity of stimulation, HN, NN, and no-stimulation (NS), was also investigated. Three steps of feature extraction, feature selection, and classification were performed to classify the stimuli. To extract the features, each trial was divided into two parts according to the stimulation onset. The first part, which includes a time interval of [−4, −1] second, was considered NS. The second part is [0, 3] seconds, which was regarded as the stimulation time interval (Figure 1F).

To extract the feature, first, theta, alpha, beta, low-gamma, and high-gamma bands were separated with Butterworth band-pass filter, and then the mean absolute value (MAV) was extracted for each sub-band (Shukla et al., 2019). Features were extracted over time from 30-millisecond non-overlapping windows, resulting in 100 features from each sub-band. Since the recording was done bilaterally, and the signals of each electrode were divided into five sub-bands, 1,000 features were extracted from each trial. Each feature was normalized by the z-score method. The role and importance of features in classification are different; therefore, the simultaneous use of all features, Increases the computational burden and may cause overfitting issues in estimating the parameters of the classification model. To avoid the mentioned issues, performing feature selection and removing irrelevant features is necessary. The minimum redundancy maximum relevance (mRMR) algorithm was used to select the best features (Max-dependency, 2005). This method is based on mutual information, which is expressed by the following equation:


(2) $$\text{I}\,\left(\text{x};\text{y}\right)=\iint \text{p}\,\left(x,y\right)\,\log\frac{p\,\left(x,y\right)}{\text{p}\,\left(\text{x}\right)\,p\,\left(y\right)}\,\text{dxdy}$$

where p(x) and p(y) are the marginal probability density functions of the x and y parameters, respectively, and p(x, y) is the joint probability distribution. The purpose of the mRMR method is, firstly, to maximize the average of the mutual information between each feature (x_*i*_) and target (y), and secondly to minimize the average of the mutual information between the two features, namelyI(x_j_; x_i_. The mRMR objective function is as follows:


(3) $$\max_{{\text{x}}_{\text{j}}\in X-{\text{S}}_{\text{k}}}\left[\text{I}\,\left({\text{x}}_{\text{j}};\text{y}\right)-\frac{1}{\text{k}}\,\sum_{{\text{x}}_{\text{i}}\in {\text{S}}_{\text{k}}}I\,\left({\text{x}}_{\text{j}};{\text{x}}_{\text{i}}\right)\right]$$

After selecting the best features by the mRMR method, stimulation was classified into three stages (HN–NS, NN–NS, and HN–NN). Numerous studies in brain signal classification have used the support vector machine (SVM) method (Xiao et al., 2019; Davoudi et al., 2021), so in this study, the SVM algorithm with Gaussian kernel was utilized for classification. SVM is a discriminative supervised learning model that delineates the classification boundary by hyperplanes with maximum margin. Specifically, SVM maps the inputs into a high-dimensional feature space and then constructs a linear optimal hyperplane in the feature space (Hosseini et al., 2010). The classification was done by two validation methods; in the first method, rats’ trials were mixed, and the results were reported by 10-fold cross-validation. In the second method, one of the rats was considered as test data, and the other rats were considered as training data (leave-one-subject-out), and these steps were repeated for all rats.

### 2.8. Phase locking value (PLV)

To evaluate the synchronization between two hemispheres, the connectivity measure of phase locking value (PLV) was exploited (Bastos and Schoffelen, 2016; Hosseini and Shalchyan, 2022). The mathematical calculation of PLV is described as follows. The continuous Morlet wavelet transform is used to calculate the complex representation of signals at various frequencies. The phases of signals from both hemispheres are then extracted based on their Morlet transformation. PLV measure at frequency *f* between two signals computed as follows (Zhang et al., 2016):


(4) $$P\,L\,V=\left|\frac{1}{N}\,\sum_{k=1}^{N}\text{exp}\,\left(i\,\left[\phi_{x}\,\left(\omega ,k\right)-\phi_{y}\,\left(\omega ,k\right)\right]\right)\right|$$

where *N* is the length of the signals. The phases of signal *x* and *y* at frequency ω are denoted by *ϕ*_*x*_ (ω, *k*) and *ϕ*_*y*_ (ω, *k*), respectively. The equation calculates the mean of phase angle differences between the two signals over time. The value of PLV is between 0 and 1. Where 0 expresses no phase synchronization and 1 is the full phase synchronization. To highlight task-related effects, the pre-trial baseline interval was subtracted from the signals. In this study, the PLV measure was used to evaluate the frequency synchronization between the left and right hemispheres.

### 2.9. Pearson’s correlation coefficient (PCC)

Pearson’s correlation coefficient (PCC) measures the linear association of two variables. Mathematically, it is defined as the ratio between the covariance of two variables and the product of their standard deviations (Risqiwati et al., 2020); thus, PCC is a normalized value between −1 and 1. PCC is formulated as follows:


(5) $$P\,C\,C=\frac{\sum_{i}\left(x_{i}-\bar{x}\right)\,\left(y_{i}-y\right)}{{\left[\sum_{i}{\left(x_{i-}\,\bar{x}\right)}^{2}\,\sum_{i}{\left(y_{i-}\,\bar{y}\right)}^{2}\right]}^{1/2}}$$

where $\bar{x}$ and $\bar{y}$ are the mean of time series $x={\left\{x\right\}}_{i=1}^{N}$, and $y={\left\{y\right\}}_{i=1}^{N}$, respectively. We used Pearson correlation to scrutinize the linear correlation between the neural activity of the two hemispheres.

### 2.10. Cross-correlation

Cross-correlation measures the linear relationship between two different signals as a function of time delay. It is generally used to quantify signals’ similarity and examine the synchronization between signals (Silva et al., 2020). The cross-correlation function between the real-valued signals *x*(*n*) and *y*(*n*) formulated as follows:


(6) $$r_{x\,y}=\frac{C_{x\,y}\,\left(k\right)}{S_{x}\,S_{y}};k=0,\pm 1,\pm 2,\ldots$$

*r*_*xy*_ is an estimate of the cross-correlation.


(7) $$C_{xy}\left(k\right)=\left\{ \begin{array}{l} \frac{1}{T}\sum_{i=1}^{T-k}(x_{i}-\bar{x})\;(y_{i+k}-\bar{y})\;;\;k=0,\;1,\;2,\;\ldots \\ \frac{1}{T}\sum_{i=1}^{T+k}(y_{i}-\bar{y})\;(x_{i-k}-\bar{x})\;;\;k=0,\;-1,\;-2,\;\ldots \end{array}\right.$$

where $\bar{x}$ and $\bar{y}$ are the sample means of the series. The sample standard deviations of the series are:


(8) $$S_{x}=\sqrt{C_{x\,x}\,\left(0\right)},w\,h\,e\,r\,e\,C_{x\,x}\,\left(0\right)=v\,a\,r\,\left(x\right).$$

(9) $$S_{y}=\sqrt{C_{y\,y}\,\left(0\right)},w\,h\,e\,r\,e\,C_{y\,y}\,\left(0\right)=v\,a\,r\,\left(y\right).$$

In this research, we used the cross-correlation method for the time lag of neural activity of the two hemispheres.

## 3. Results

### 3.1. Rats’ behavioral responses

In animal studies, pain is not directly measurable, so rat behavioral responses are used to measure pain intensity (Deuis et al., 2017). Behavioral responses to nociceptive pain include shaking the head, moving the body, lifting, pulling, and licking the foot (Fan et al., 1995). In the current study, the intensity of stimuli was selected based on the behavioral response of the rats. At the beginning of each NN session, 10 to 15 stimuli were first applied to make the animals accustomed to the experimental environment and reduce their stress and fear and then the main part of the NN sessions started. In NN stimulations, the duration of the NN stimulation period was 5 s, that is, the filament was in contact with the rat’s hind paw for 5 s. Since the intensity of the stimulation was less than the pain threshold value, the rats usually did not respond to the stimulation during this period. To further ensure the validity of the NN results, trials in which rats showed a painful response (shaking, moving, lifting, pulling, and licking) were discarded as invalid trials. The animals during NN sessions withdrew their paw in less than 3% of trials. These trials were removed from the analysis. In other words, the stimulus did not lead to a painful response in NN sessions. The time interval from the onset of stimulation to the moment of paw withdrawal was reported based on mean ± SEM. In HN stimulation to the hind paw, the rats lifted their paw with a delay of 225 ± 10 ms. Lifting the paw was sometimes accompanied by licking and gentle bites. According to the rats’ reaction to stimuli, it can be said that filament 8 g was non-noxious, and filament 30 g was noxious for all rats. Also, reaction time from session to session and rat to rat was examined. The results showed that the reaction time significantly differed from rat to rat; however, no significant pattern was observed from session to session.

### 3.2. Evoked potentials analysis

**FIGURE 2:**
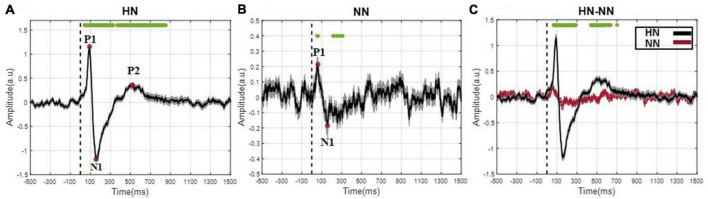
The evoked potential was displayed as mean ± SEM. **(A)** The evoked potentials are related to HN stimulation. The difference between each time sample of HN stimulation interval and baseline interval was statistically evaluated, and samples with a significant difference are denoted by green dots (Wilcoxon signed rank test with a *p*-value < 0.05). **(B)** The evoked potentials related to NN stimulation (Wilcoxon signed rank test with a *p*-value < 0.05). **(C)** The evoked potentials related to NN and HN stimulations are superimposed in a plot (Wilcoxon rank sum test with a *p*-value < 0.05). Time 0, demonstrated by the dashed line, represents the onset of mechanical stimulation.

After removing the motion and mains artifact by the Butterworth filter and removing defective trials, 657 HN and 533 NN stimuli were used for processing. Although pooling data from different animals and sessions is a potentially dangerous practice, by examining the signals of each rat in each session, the risk of “grand average” was minimized as much as possible. According to Figure 2A, the EP of HN stimulation for 1 s after stimulation was also significantly different from the baseline. In NN stimulation, a significant difference occurred in a shorter and interrupted interval (Figure 2B). The EPs in the HN and NN stimuli had a similar pattern. However, they differed in the amplitude and timing of the positive peaks and negative peaks. Statistical tests were used to compare the EPs of the two stimuli over time more precisely. The results show that during EPs, the signal amplitudes after two stimuli were significantly different (Figure 2C). Domain parameters and the occurrence times of P1 and N1 were extracted to investigate the stimulus pattern, and the results are reported in Table 1.

**TABLE 1:** Demonstration of significant difference between HN and NN stimulation parameters with Wilcoxon rank-sum test.

Evoked potential parameters	HN	NN	Statistical test
The time interval between P1 and the stimulation onset time (ms)	92 ± 2.26	61 ± 3.82	*p* < 10^−5^
The time interval between N1 and the stimulation onset time (ms)	157 ± 2.59	157 ± 3.89	*p* = 0.81
P1 amplitude	1.15 ± 0.04	0.21 ± 0.04	*p* < 10^−5^
N1 amplitude	−1.17 ± 0.04	−0.18 ± 0.04	*p* < 10^−5^

The results were expressed based on mean ± SEM.

The time of the P1 in NN stimulation is significantly less than in HN stimulation. However, the N1 in the two stimuli occurred almost with similar delay, so it can be said that the time interval between P1 and N1 in HN stimulation is shorter than in NN. In HN stimulation, the amplitude of P1 was 5.47 times that of NN stimulation, and this ratio was 6.5 for the N1 valley. In HN stimulation, the P2 was observed, which was not observable clearly in NN (Table 1). According to the mentioned cases, it can be said that the pattern of variations of EPs in two stimulations are almost similar, but they are different in the amplitude and time of occurrence of peaks and valleys.

### 3.3. Power variations in different bands

Local field potential (LFP) provides information about the collective behavior of neural groups. Different frequency bands represent the activity of these neurons; for example, the power of high-frequency LFP (gamma) can be an indirect representation of spikes generated by nearby neurons (Buzsáki et al., 2012). So it is essential to study the LFP signals in the frequency domain. In this study, the Morlet wavelet transform has been used to investigate the time-frequency domain. The power of each frequency was normalized to its baseline value to illustrate the power changes more clearly. The time interval selected for the baseline was [−1, 0] seconds. Each extracted frequency was normalized to its baseline by the z-score method (Cohen, 2019).

**FIGURE 3:**
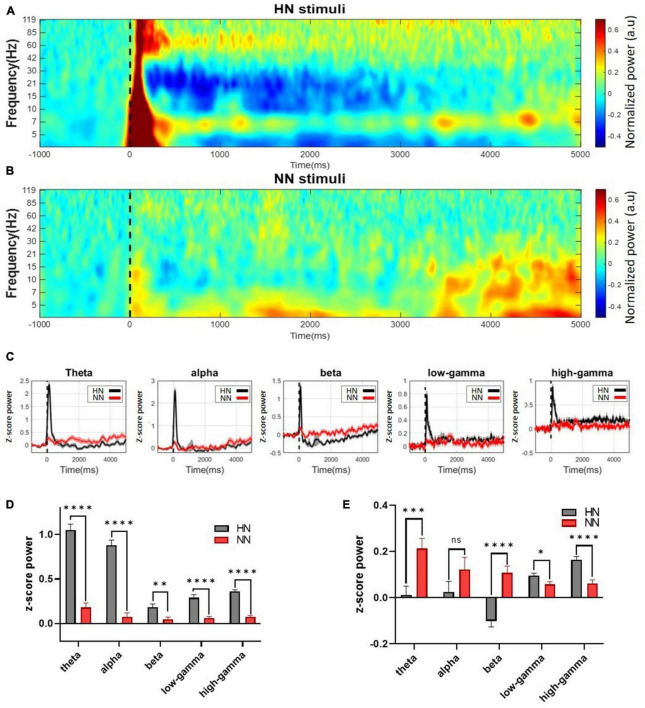
Continuous wavelet transform with Morlet mother wavelet was used to extract the power. Mechanical stimuli are applied at time 0, which is shown by a dashed line. **(A)** The power of the HN signal in the time-frequency domain. **(B)** The power of the NN signal in the time-frequency domain. Power variations in this stimulation occurred in a shorter interval and a more limited frequency band than HN stimulation. **(C)** Continuous normalized powers calculated in different frequency bands. **(D)** The power of the signal in different frequency bands was calculated in 0–500 ms intervals after HN and NN stimulation compared to baseline power. **(E)** Power of frequency bands between 500 ms and 5,000 ms. ^n.s.^(*P*-value > 0.05), *(0.01 < *P*-value < 0.05), **(0.001 < *P*-value < 0.01), ***(0.0001 < *P*-value < 0.001), ****(*p*-value < 0.0001) Wilcoxon rank-sum test.

In HN stimulation, power increased visibly from 3 to 120 Hz immediately after stimulation. After 500 ms of HN stimulation, the power changes varied at different frequencies. At frequencies of 30 to 120 Hz, the amount of power was still higher than the baseline value for about 5 s after stimulation. Nevertheless, at frequencies 8–30, the amount of power was reduced to less than the baseline. This power reduction persisted for up to 5 s after stimulation onset (Figure 3A). In NN stimulation, power changes were also noticeable, but these changes were less severe than HN stimulation (Figure 3B).

Statistical tests should be used to make definitive statements about power changes. Signals were divided into five sub-bands (4–8 Hz theta, 8–12 Hz alpha, 12–30 Hz beta, 30–80 Hz low-gamma, and 80–120 Hz high-gamma) to examine the frequency activities in more detail (Figure 3C). To further investigate power changes, power was extracted at intervals of [0, 0.5] seconds (EPs interval) and [0.5, 5] seconds (after EPs interval). Significant differences between the power of LFPs after HN and NN stimulation were determined using the rank-sum test. In the interval of [0, 0.5] seconds, the power of all sub-bands was significantly increased in proportion to the intensity of stimulation (Figure 3D). Power changes persisted after the EPs interval. However, the behavior of the changes was different. It was observed that in the interval of 0.5–5 s, the theta and beta bands with increasing stimulation intensity, the power decreased significantly. In low-gamma and high-gamma bands, the power was significantly increased, directly related to the stimulation intensity (Figure 3E).

### 3.4. Classification performance

Local field potentials (LFPs) in the ACC encode information about the intensity of stimulations. In the previous section “3.3. Power variations in different bands,” the power of the LFP sub-band was used to investigate the difference between the two stimulation. However, other features can also demonstrate the separability of the three states, HN, NN, and NS, in more detail. A total of 657 HN trials and 533 NN trials, and 1,190 NS trials were used for classification. One thousand features were extracted from each trial. After choosing the best features by the mRMR algorithm, classification was done. Two validation approaches performed classification.

**FIGURE 4:**
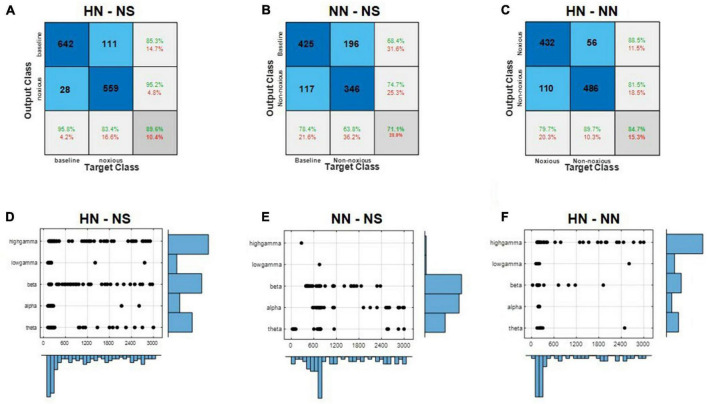
Local field potential (LFP) decoding analysis using supervised machine learning predicts the intensity of pain. **(A–C)** Demonstrate the HN–NS, NN–NS, and HN–NN classification confusion matrices, respectively. **(D–F)** Show the distribution of selected features by the mRMR method in time and frequency for classifying HN–NS, NN–NS, and HN–NN, respectively.

In the first method, all rats’ trials were combined, and the results were reported by 10-fold cross-validation. In the HN–NS classification, the best accuracy was achieved with 123 features. The best features were generally selected from the high-gamma and beta bands in the time interval of 0–500 ms (Figure 4D). The SVM method could reliably distinguish these two classes with 89.6% accuracy and kappa = 0.79 (Figure 4A). The area under the ROC curve (AUC) was 0.94, which shows that this classifier could separate the two classes with outstanding results (Mandrekar, 2010). In the NN–NS classification, 74 features were selected that were generally from the alpha and beta bands in the initial 1,200 ms (Figure 4E). According to the confusion matrix, the SVM method could separate these two classes with acceptable results (accuracy = 71.1%, kappa = 0.41, and AUC = 0.71) (Figure 4B). In the NN–HN classification, 57 features were selected as the best features. Best features were generally selected from the high gamma band in the time interval 0–600 ms (Figure 4F). The two stimuli were separated with 84.7% accuracy and kappa = 0.69 (Figure 4C). The AUC value was 0.91, which demonstrates that this classifier could separate these two stimuli with outstanding results. The best features for discriminating the intensity of stimulation were generally selected in the EP occurrence time interval, the gamma and beta bands are the most effective bands in differentiating pain intensities, respectively (Figures 4D–F).

In the second method, leave-one-subject-out was used. The algorithm was repeated 100 times for each rat in this method, and then the results were reported (Table 2). HN stimulation was separated from NS with 88.79% accuracy, which was 1% lower than the previous method. NN stimulations were distinguished from NS with 71.65% accuracy, which was not significantly different from the former method. HN stimulation was separated from NN with 79.04% accuracy, which was 5.5% lower than the previous method. Given that the results of leave-one-subject-out and 10-fold cross-validation were close to each other, we can say that the processing methods are comprehensive and can be generalized to new rats.

**TABLE 2:** The classification results are reported based on the leave-one-subject-out method.

	HN and baseline		NN and baseline		NN and HN	
	**Accuracy (%)**	**Kappa**	**Accuracy (%)**	**Kappa**	**Accuracy (%)**	**Kappa**
Rat1	90.70	0.81	78.79	0.57	75.33	0.49
Rat2	81.19	0.62	77.24	0.54	67.05	0.33
Rat3	91.37	0.82	72.07	0.44	83.22	0.63
Rat4	84.86	0.69	66.72	0.33	70.05	0.39
Rat5	97.54	0.95	67.16	0.34	92.16	0.83
Rat6	91.67	0.83	68.87	0.37	79.70	0.59
Rat7	84.23	0.68	70.70	0.41	86.13	0.71
Mean	88.79 ± 5.6	0.77 ± 0.11	71.65 ± 4.7	0.42 ± 0.09	79.04 ± 8.9	0.56 ± 0.17

### 3.5. Interhemispheric neural characteristics

**FIGURE 5:**
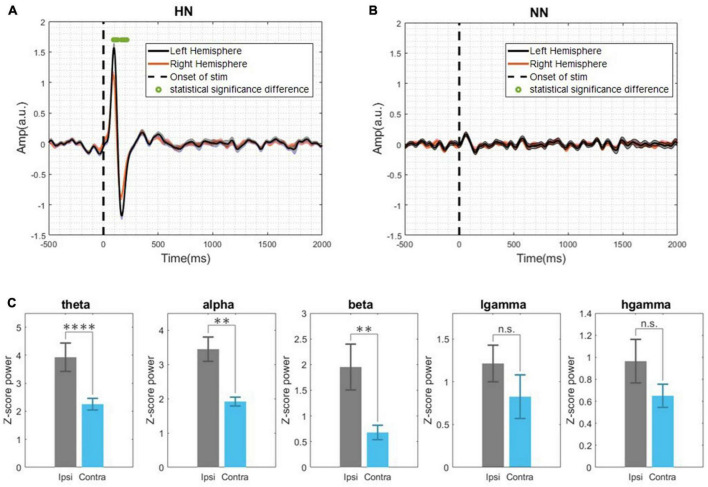
**(A)** Demonstrates the evoked potential of HN stimulation bilaterally and examines the statistical differences between the two hemispheres during the time interval [–0.5, 2] s. **(B)** NN evoked potential in two hemispheres. The Wilcoxon signed rank test with a 95% confidence interval was used to determine the significance of differences. **(C)** Band power in the interval of [0–500] ms and HN stimulation (Ipsilateral and contralateral). ^n.s.^(*P*-value > 0.05), **(0.001 < *P*-value < 0.01), ****(*p*-value < 0.0001) Wilcoxon signed rank test.

By investigating the EP of two hemispheres, we found that, in NN stimulation, there was no significant difference between EPs in the two hemispheres (Figure 5B). In HN stimulation, the absolute amplitude of P1 and N1 in the ipsilateral was significantly higher than the contralateral (*p* <  10^−5^, Wilcoxon signed-rank test) (Figure 5A). However, in NN there was no significant difference. Latencies of P1 and N1 in the two hemispheres were not significantly different (*p* > 0.05, Wilcoxon signed-rank test). The LFP band power of two hemispheres in HN and NN was investigated in two-time intervals ([0–500] ms and [0.5–5] seconds). Investigating the signal power between the two hemispheres showed that the LFP power was significantly different in the two hemispheres only in HN stimulation. The power of the theta, alpha, and beta bands on the ipsilateral hemisphere was significantly higher than the contralateral in the interval of 0–500 ms (Figure 5C). However, no significant difference between LFP band powers in the two hemispheres was observed in any stimulation, in the 0.5–5 s interval.

**FIGURE 6:**
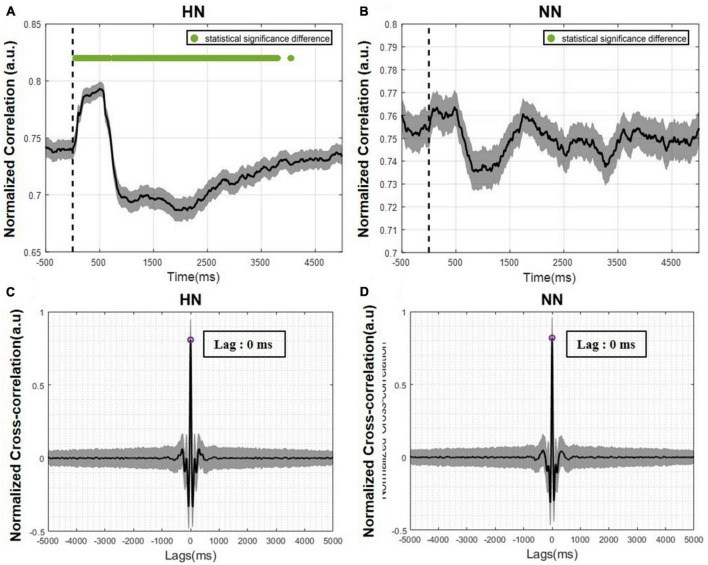
The correlation between the two hemispheres over time is plotted as mean ± SEM. The statistical test results were calculated with a *p*-value < 0.05. **(A)** Correlation of the two hemispheres over time in HN stimulation. **(B)** Correlation of the two hemispheres over time in NN stimulation. The Wilcoxon signed rank test with a 95% confidence interval was used. **(C)** Cross-correlation between the two hemispheres in HN stimulation. **(D)** Cross-correlation between the two hemispheres in NN stimulation.

To investigate the activity synchronization between two hemispheres, a cross-correlation analysis was performed. Cross-correlation measures the synchronization between two signals by time lagging one of them and calculating the correlation between them as a function of time lag. To calculate the time lag between hemispheres, first, the cross-correlation in each trial was calculated, and finally, the average of all trials was determined. The time lag between two hemispheres’ activity is equivalent to the time that the greatest correlation occurred. Cross-correlation peaked in both HN and NN stimulations at time = 0 (Figures 6C, D). This result shows that the activity of the two hemispheres in ACC is synchronous.

The correlation over time was calculated to examine the LFP signal in the two hemispheres more accurately. For this purpose, the correlation between the two hemispheres in each trial was calculated using a 500-ms sliding window with a 1-ms step and averaged over all trials. In HN stimulation, immediately after stimulation, the correlation between the two hemispheres increased significantly and then decreased. This decrease was so great that it became lower than the baseline value (Figure 6A). These correlation variations persisted for up to 4 s after stimulation and then returned to pre-stimulation values. In NN stimulation, the correlation changes were like HN stimulation, but these changes were not significant, and returning to the baseline level occurred sooner than in HN stimulation (Figure 6B).

**FIGURE 7:**
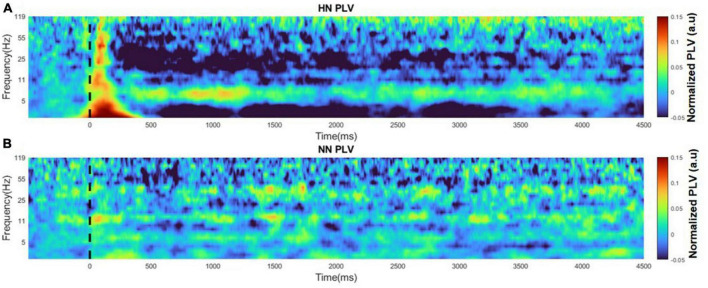
Time-frequency plot of PLVs for HN and NN sessions. The figures are normalized to baseline activity. **(A)** PLV time-frequency plot for HN stimulation. **(B)** PLV time-frequency plot for NN stimulation.

Generally, connectivity measures the interaction between different regions. Correlation connectivity represents the similarity of two regions from an amplitude domain perspective. To study the phase difference between the two hemispheres, we employed the PLV analysis. In this study, PLV was calculated over time and frequency using continuous wavelet transform. Variations in PLV after stimulation were various in different bands. After applying the HN stimulus, the two-hemisphere PLV increased in all bands in the EP interval. After about 500 ms of stimulation, PLV continuously decreased to less than the baseline value. The reduction of PLV is more evident in the beta and low-gamma bands. The recovery period was non-identical in different bands. In addition, at low frequencies, the PLV recovery cycle was longer than the high frequencies. The duration of variations in HN stimulation lasted approximately 4 s (Figure 7A). PLV fluctuations between the two hemispheres are not informative in NN sessions (Figure 7B).

In general, it seems that mechanical stimulation affects the phase synchronization between the two hemispheres of the ACC area, and the greater the stimulation intensity, the greater the duration time, and intensity of the oscillations.

## 4. Discussion

The ACC response to mechanical stimuli in time and frequency was investigated in the present study. Previous studies have mostly examined the brain signal induced by thermal stimulation (Iannetti et al., 2008; Frot et al., 2016), and usually, the recording was done unilaterally. To the best of our knowledge, this is the first study investigating the effect of mechanical stimulation in the ACC regions bilaterally. We evaluate the neuronal activity of the ACC region from the perspective of EP analysis, power analysis, stimulation intensity classification, and connectivity of two hemispheres. The main goal of this study is to investigate the persistence of the pain effect in brain signals and examine the interactions between the two hemispheres. As far as we know, this is the first study that examines the correlation and phase lag of the two hemispheres over time. Also, another innovation of this research was the investigation of variations in power, EPs, correlation of two hemispheres, and the phase lag of two hemispheres over time to check the persistence of pain effect on brain signals.

Evoked potential (EP) wave latency and amplitude demonstrate, respectively, the length of time spent and the number of neural resources participating during information processing (Lin et al., 2019). Therefore, neural correlates of EP waves were specially investigated in detail. To extract EPs, 657 HN and 533 NN trials were averaged from seven Wistar rats for a valid EP waveform. The pattern of EPs variations in the two stimuli was similar but differed in amplitude and latency of EPs waves. It was observed that the amplitude of P1, N1, and P2 increased significantly with increasing intensity of mechanical stimulation. It was also concluded that the stimulation intensity has a direct relationship with the P1 latency; that is, with the increase of the intensity, the P1 latency increased significantly. However, the two stimuli had no significant difference in the N1 latency. Reviewing previous studies showed that the pattern of EP in thermal stimulation (Li et al., 2017; Zhang et al., 2018) was similar to mechanical stimulation.

Pain intensity classification was done in previous studies, but the stimuli were generally thermal (Wang et al., 2003, 2008; Zhang et al., 2011, 2018; Li et al., 2017; Urien et al., 2018; Xiao et al., 2019). In the present work, the intensities of mechanical stimuli were classified. A recent study used both the spike and LFP features to classify laser stimulations, which reported 80% classification accuracy between HN and NN stimulation (Zhang et al., 2018). In another study, data from four regions, ACC, S1, orbitofrontal cortex (OFC), and periaqueductal gray (PAG), were used to classify HN and NN thermal stimuli. They achieved an accuracy of 86% (Li et al., 2017). In our study, only LFP features were used for classification. The classification results were analyzed by leave-subject-out and 10-fold cross-validation methods. Classification accuracies based on the 10-fold cross-validation method in HN–NS, HN–NN, and NN–NS scenarios were 89.6, 84.7, and 71.1%, respectively. The results of the leave-one-subject-out method were close to the 10-fold results, which shows the comprehensiveness and generalizability of the model. In addition, based on the feature selection analysis, we showed which time interval after stimulation and which frequency bands are more effective in distinguishing pain intensity (Figure 4). In noxious stimulation, features were generally selected from the [0 to 500] ms interval and gamma and beta bands. However, in non-noxious stimulation, the best features were selected from [500 to 1,000] ms and alpha and beta bands.

In the brain, the experience of pain is associated with neuronal oscillations at frequencies ranging from infra-low fluctuations to high-frequency oscillations (Ploner et al., 2017). Painful stimulation creates distinct time and frequency patterns in brain signals. A previous study noted that the ACC region’s alpha and beta band power decreases due to painful thermal stimulation, and the gamma band power increases (Li et al., 2017). Another study showed that theta and high-gamma band power increased significantly due to painful thermal stimulation (Zhang et al., 2018). In this study, power was extracted during the EP occurrence interval (0–0.5 s) and after the EP occurrence interval (0.5–5 s) using continuous Morlet wavelet transform. The power of all bands increased significantly during the EP interval, and the power was directly related to stimulation intensity. However, the band power variations were different in the post-EP interval. During this interval, low frequencies (theta, alpha, and beta) had an inverse relationship with stimulation intensity. Whereas high frequencies (low-gamma and high-gamma) had a direct relationship with stimulation intensity. Power variations persist for up to 5 s after stimulation, which indicates the permanence of the noxious stimulation effect in the ACC regions. The power analysis findings corroborate the classification results. It mainly showed that high gamma, beta, and theta band power activities represent the most discrimination between HN and NN stimulations. In fact, the power variations of these bands have gone through a more stable process with fewer fluctuations until the return to the baseline activity (Figure 3C).

In a previous related study to evaluate the brain function caused by thermal stimulation, electrodes were implanted bilaterally in S1, ventral posterior thalamus (VP), ACC, and medial dorsal thalamus (MD) regions. Their result showed that the medial pain pathway (ACC, MD) had bilateral, faster, and smoother responses. However, in the lateral pain pathway (S1, VP), the responses were transient and delayed, and these regions were activated contralaterally (Wang et al., 2003). In more detail, our study investigated the similarities and differences of EPs, correlation, and PLV in the two hemispheres. The results showed that the EPs in the two hemispheres were very similar and had the same pattern. However, in HN, the amplitudes P1 and N1 on the ipsilateral side were significantly greater than contralateral. In addition, our results show that EPs occur simultaneously in both hemispheres. Furthermore, the analysis of the correlation between two hemispheres showed that in HN stimulation, the correlation value changes after applying the stimulus, and after about 4 s, the correlation value returns to the pre-stimulation value. Perhaps this 4-s interval is related to the persistence of the pain effect. Correlation variations over time in NN stimulation were not significantly different from baseline activity. To further investigate the relationship between the two hemispheres, the PLV of the two hemispheres over time was examined. The results showed that the stimulation intensity was directly correlated with the PLV variations period. In other words, the more intense the stimulation, the longer it takes for PLV to return to the baseline value.

## 5. Conclusion

In a nutshell, the objective of this research was twofold. First, we aimed to investigate the discrimination of mechanical stimulations from ACC brain recordings in a rat model. Our results based on EP, power, and time-frequency analysis revealed that mechanical stimulations at different intensities can be distinguishable from ACC activities. The second goal was to study the activity of the two hemispheres from amplitude and phase perspectives at various frequencies and time intervals. We showed that the ACC is activated bilaterally. The variations found in correlation and PLV analysis of both hemispheres would persist for a few seconds after stimulation onset. Although the presence of noxious mechanical stimulus was less than 250 ms, the effect of mechanical pain would persist for up to 4 s after stimulation. This claim is supported based on bilateral desynchronization activities via correlation and PLV connectivity analysis. The power analysis also revealed that the variations in power would return to baseline value after about 4 s corroborating the results found in bilateral connectivity analysis.

## 6. Limitations

In this work, only one area was recorded bilaterally. Although, increasing the areas of implantation can lead to the examination of brain connectivity in more detail. Also, it is possible to use electrodes that can record neural activity at spike and LFP levels. In the task design part, by increasing the variety of stimulation intensity and also applying stimulation to both paws, allows us to examine the effects of mechanical stimulation on the brain signal in more detail.

Also, motor activity is an integral part of pain-related tasks in freely moving experiments, therefore, due to the proximity of the ACC region and the motor cortex, there is concern about interference in neuronal activity. In this research, we tried to minimize the effects of movement by removing the trials that had long movements and examining the activity of neuronal activity in two intervals.

## Data availability statement

The raw data supporting the conclusions of this article will be made available by the authors, without undue reservation.

## Ethics statement

This animal study was reviewed and approved by the Animal Care and Use Committee of Neuroscience and Neuroengineering Research Laboratory, Iran University of Science and Technology.

## Author contributions

AA and VS conceived the initial research idea and designed the research. AA performed the electrode constructions, hardware, and software implementations, data analysis, and wrote the manuscript. AA and MM performed all animal experiments and data recordings. MG helped in animal surgeries and critically reviewed the manuscript. VS supervised the experiments, analyzed and interpreted the results, and reviewed and revised the manuscript. All authors contributed to the article and approved the submitted version.

## Footnotes

1
https://arriveguidelines.org
